# A Flight Back to Ground: Jung’s Recalcitrant Fourth as Rape into Consciousness. Symbolic Rape and Literal Rape in Persephone’s Myth

**DOI:** 10.1111/1468-5922.13106

**Published:** 2025-06-25

**Authors:** Barbara Cerminara

**Affiliations:** ^1^ Norwich UK

**Keywords:** gender‐based violence, *Hymn to Demeter*, October 7^th^, Persephone, rape, recalcitrant fourth, viol, violence basée sur le genre, 7 octobre, Perséphone, quatrième récalcitrante, Hymne à Déméter, Vergewaltigung, geschlechtsspezifische Gewalt, 7. Oktober, Persephone, das widerspenstige Vierte, *Hymne an Demeter*, sturpo, violenza di genere, 7 ottobre, Persefone, quarto recalcitrante, *Inno a Demetra*, изнасилование, гендерное насилие, 7 октября, Персефона, бунтарское четвертое, «Гимн Деметре», violación, violencia‐de‐género, 7 de Octubre, Perséfone, cuatro recalcitrante, Himno de Deméter, 强奸, 基于性别的暴力, 10月7日, 珀耳塞福涅, 顽固的第四, 德墨忒尔颂歌

## Abstract

Patricia Berry’s interpretation of the Demeter/Persephone myth, and her concept of *rape into consciousness*, illuminate intrapsychic dynamics. However, this symbolic lens may inadvertently distance us from the devastating nature of literal rape—a reality the Homeric *Hymn* encapsulates. Jung’s concept of *recalcitrant fourth* offers a crucial counterpoint, demanding a shift from the symbolic to the empirical world. This violent flight back to ground forces a confrontation with life’s material and sensorial dimensions, where literal rape reaps trauma and death. Jung’s fourth, reconceptualized here as rape into consciousness, compels the retrieval of the *neglected*: elements absent from conscious awareness. Guided by this concept and the Persephone myth, the author examines rape’s concrete realities, urging witness to its traumatic devastation. The Persephone/Demeter myth, in Jung’s view, reflects a split between matriarchal and patriarchal worlds. The Homeric *Hymn* intensifies this fracture, bringing into full view the violence perpetrated by the patriarchy against the two female protagonists. Played within the divine realm, this archetypal gender conflict reverberates throughout history, appearing in different forms, from the personal sphere to the horrors of war and terror. The events of October 7^th^ serve as a grim reminder of its enduring presence.

## Abstract (fr)

L'interprétation du mythe de Déméter/Persephone par Patricia Berry, ainsi que son concept de viol dans la conscience, éclairent les dynamiques intrapsychiques. Cependant, cette lentille symbolique peut nous éloigner involontairement de la nature dévastatrice du viol littéral—une réalité que l'Hymne homérique encapsule. Le concept de quatrième récalcitrante de Jung offre un contrepoint crucial, exigeant un passage du symbolique au monde empirique. Cette fuite violente vers le concret force une confrontation avec les dimensions matérielles et sensorielles de la vie, où le viol littéral récolte traumatisme et mort. Le quatrième de Jung, reconceptualisé ici en tant que viol dans la conscience, oblige à la récupération de ce qui est négligé : les éléments absents de la conscience. Guidé par ce concept et le mythe de Perséphone, l'auteur examine les réalités concrètes du viol, incitant à faire témoignage à la dévastation traumatique qui s’en suit. Le mythe de Perséphone/Déméter, selon Jung, reflète une division entre les mondes matriarcaux et patriarcaux. L'Hymne homérique intensifie cette fracture, mettant en pleine lumière la violence perpétrée par le patriarcat contre les deux protagonistes féminines. Joué dans le domaine divin, ce conflit de genre archétypal résonne à travers l'histoire, apparaissant sous différentes formes, de la sphère personnelle aux horreurs de la guerre et du terrorisme. Les événements du 7 octobre 2023 servent de rappel sinistre de sa présence persistante.

## Abstract (de)

Patricia Berrys Interpretation des Demeter‐/Persephone‐Mythos und ihr Konzept der *Vergewaltigung ins Bewußtsein* beleuchten intrapsychische Dynamiken. Diese symbolische Sichtweise kann uns jedoch unbeabsichtigt von der verheerenden Natur der buchstäblichen Vergewaltigung distanzieren – einer Realität, die der Homerische *Hymnus* beinhaltet. Jungs Konzept der *widerspenstigen Quarte* bietet einen entscheidenden Kontrapunkt und erfordert einen Wechsel von der symbolischen zur empirischen Welt. Diese gewaltsame Flucht zurück auf den Boden der Tatsachen erzwingt eine Konfrontation mit den materiellen und sensorischen Dimensionen des Lebens, wo buchstäbliche Vergewaltigung Trauma und Tod nach sich zieht. Jungs Viertes, hier als bewußte Vergewaltigung neu konzipiert, zwingt dazu, das *Vernachlässigte* wiederzuentdecken: Elemente, die im Bewußtsein fehlen. Geleitet von diesem Konzept und dem Persephone‐Mythos untersucht die Autorin die konkrete Realität der Vergewaltigung und fordert dazu auf, deren traumatische Folgen zu bezeugen. Der Persephone/Demeter‐Mythos spiegelt für Jung eine Spaltung zwischen matriarchaler und patriarchaler Welt wider. Der Homerische *Hymnus* verschärft diesen Bruch und bringt die Gewalt des Patriarchats gegen die beiden Protagonistinnen deutlich zum Ausdruck. Dieser archetypische Geschlechterkonflikt, der sich im göttlichen Reich abspielt, hallt durch die Geschichte und tritt in verschiedenen Formen in Erscheinung, vom Persönlichen bis hin zu den Schrecken von Krieg und Terror. Die Ereignisse vom 7. Oktober 2023 erinnern eindringlich an seine anhaltende Präsenz.

## Abstract (it)

L’interpretazione che Patricia Berry ha dato del mito di Demetra/Persefone, e il suo concetto dello *stupro nella coscienza*, illumina le dinamiche intrapsichice. Eppure, questa lettura simbolica può, inavvertitamente, allontanarci dalla natura devastante dello stupro concreto —una realtà che *l’Inno* omerico ha incapsulato. Il concetto di Jung del *quarto recalcitrante* ci offre un contrappunto fondamentale, richiedendo un passaggio dal mondo simbolico a quello reale. Questo violento ritorno sulla terra ci obbliga a confrontarci con le dimensioni materiali e sensoriali della vita, dove lo stupro concreto porta con sè trauma e morte. Il quarto recalcitrante di Jung, qui riconcettualizzato come uno stupro nella coscienza, richiede il recupero di ciò che è stato *trascurato*: gli elementi assenti dalla consapevolezza della coscienza. Guidata da questo concetto e dal mito di Persefone, l’Autrice prende in considerazione la realtà concreta dello stupro, che reclama testimonianza per il suo impatto devastante. Il mito di Persefone/Demetra, nella visione di Jung, riflette la scissione tra i mondi del matriarcato e del patriarcato. *L’Inno* omerico enfatizza questa scissione, mettendo bene in luce la violenza perpetrata dal patriarca sulle due protagoniste femminili. Avendo luogo nel dominio divino, questo conflitto archetipico si riverbera nella storia, e si mostra in forme differenti, dalla sfera personale agli orrori della guerra e del terrore. Gli eventi del 7 ottobre 2023 ci ricordano dolorosamente questa persistente realtà.

## Abstract (ru)

Интерпретация Патрицией Берри мифа о Деметре и Персефоне и ее идея о насильственном проникновении в сознание проливают свет на внутрипсихическую динамику. Однако эта символическая линза может непреднамеренно увести нас в сторону от разрушительной природы буквального изнасилования — реальности, воплощенной в гомеровском гимне. Юнговский бунтарский четвертый элемент вносит критический контраргумент и требует перехода из символического мира в эмпирический. Насильственное возвращение на землю вызывает конфронтацию с материальными и чувственными измерениями жизни, когда буквальное насилие ведет к травме и смерти. Юнговское четвертое, переосмысленное здесь как «насильственное проникновение в сознание», требует возвращения пренебрегаемого – элементов, отсутствующих в сознании. Руководствуясь концепцией четверицы и мифом о Персефоне, автор исследует буквальные реалии насилия и призывает читателей быть свидетелями его опустошительного разрушения. Миф о Персефоне и Деметре, по мнению Юнга, отражает расщепление между матриархальным и патриархальным мирами. Гомеровский «Гимн» усиливает это расщепление, наглядно демонстрируя насилие, вершимое патриархатом в отношении двух главных героинь. Архетипический гендерный конфликт, разыгрываемый в божественном царстве, продолжается на протяжении всей истории, проявляясь в различных формах, от личной судьбы до ужасов войны и терроризма. События 7 октября 2023 года служат мрачным напоминанием о его постоянном присутствии.

## Abstract (es)

La interpretación de Patricia Berry del mito de Deméter/Perséfone, y su concepto de violación en la consciencia, iluminan las dinámicas intrapsíquicas. Sin embargo, esta lente simbólica puede distanciarnos inadvertidamente de la naturaleza devastadora de la violación literal, una realidad que el Himno Homérico encapsula. El concepto de Jung del cuatro recalcitrante ofrece un contrapunto crucial, exigiendo un cambio desde el mundo simbólico al empírico. Este violento retorno a los fundamentos fuerza una confrontación con las dimensiones materiales y sensoriales de la vida, donde de la violación literal se obtienen trauma y muerte. El concepto de Jung del cuatro recalcitrante, reconceptualizada aquí como violación de la conciencia, obliga a recuperar lo olvidado: elementos ausentes de la percepción consciente. Guiada por este concepto y por el mito de Perséfone, la autora examina las realidades concretas de la violación, instando a dar testimonio de su devastación traumática. Desde la perspectiva de Jung, el mito de Perséfone refleja una escisión entre los mundos matriarcal y patriarcal. El Himno Homérico intensifica esta fractura, trayendo a la luz la violencia perpetrada por el patriarcado contra las dos protagonistas femeninas. Interpretado en el ámbito divino, este conflicto arquetípico de género reverbera a lo largo de la historia, apareciendo bajo diferentes formas, desde la esfera personal hasta los horrores de la guerra y el terror. Los sucesos del 7 de octubre de 2023 sirven a modo de sombrío recordatorio de su perdurable presencia.

## Abstract (zh)

帕特里夏·贝里对德墨忒尔/珀耳塞福涅神话进行了解读, 并提出了“意识的强奸”的概念, 这揭示了一个内在的心理动力。然而, 这种象征性的视角可能会无意中让我们远离字面意义上强奸的毁灭性本质——而《荷马颂歌》正是对这一现实的浓缩。荣格的“顽固第四”概念提供了一个关键的反向视角, 要求我们从象征世界转向经验世界。这种暴力的回归大地迫使我们直面生活的物质和感官维度, 而字面意义上的强奸在这里造成了创伤和死亡。荣格的第四元素, 在这里被重新概念化为“意识的强奸”, 它迫使我们重新找回那些被忽视的、意识中缺失的元素。在这一概念和珀耳塞福涅神话的指引下, 作者审视了强奸的具体现实, 呼吁见证其创伤性毁灭。在荣格看来, 珀耳塞福涅/德墨忒尔神话反映了母系社会与父系社会之间的分裂。《荷马颂歌》加剧了这一断裂, 将父权制对两位女性主角施加的暴力完全暴露出来。这一在神界上演的原型性别冲突在历史上不断回响, 以不同的形式出现在个人领域, 甚至也出现在战争和恐怖主义的恐怖领域中。2023年10月7日的事件是一个令人不快的提醒, 表明这种冲突的持久存在。

## Introduction

This paper is concerned with literal rape, with particular emphasis on the rape of women at the hands of men in conflict situations. While men can also be victims of sexual violence in war, the focus on women in this paper is warranted by the unique and devastating impact that aggravated gender‐based violence has on the female body, a trauma which transcends the individual victims involved.

As a profound invasion of the woman’s body, the act of rape mirrors the broader invasion of her homeland (Levenkron, 2010). Moreover, the brutal assault on the female reproductive apparatus creates a catastrophic disruption of the woman’s ethnic lineage, effectively seizing the future generations of the targeted community (Gaca, 2010).

I write in particular for the young women slaughtered and abducted on October 7^th^, 2023. These incidents serve as the latest manifestations of *rape culture*, a pervasive and tragic narrative woven throughout human history, and vividly reflected in mythology. Naturally, I hold in mind the many others who have been used as weapons of war, suffering abduction, sexual enslavement, and death. I am thinking of the Yazidi women captured in Iraq, and those taken in Nigeria, or the Rohingya women in Myanmar, or the Uighur women of China, the women of Rwanda and Ukraine, the women of Bosnia, the women and girls of Sudan, or those of the Democratic Republic of Congo which, according to feminist writer Pat Barker, is the “the rape capital of the world” (cited in Judge, 2024, p. 24). The list sadly goes on.

Finally, I write for the countless women enduring sexual violence within the confines of their domestic lives, where their existence has been transformed into a voiceless battleground.

Moving between 21^st^ century events, Greek myth, Nobel Laureate Louise Glück’s (2021) poetry, trauma research, and research on ancient warfare, I call the reader to bear witness as I delve into the trauma of aggravated gender‐based violence. The thread that seams the various sections of this paper together is the myth of Persephone, as narrated in the Homeric *Hymn to Demeter*: her abduction, rape, and murder.

There are different ways of reading this myth, and indeed scholars agree that the “meaning of Persephone is determined by the era in which her story is told” (Horbury, 2015, p. 3). A branch of modern scholarship interprets Persephone’s rape as an initiation into womanhood (see, for example, Bolen, 1984; Jennifer & Roger Woolger, 1987; Richardson, 2011; Foley, 2013; Lincoln, 2013; Bushe, 2013). In this paper I read the story of Persephone merely as a tale of trauma: Persephone, I suggest, suffers from the compulsion to repeat typical of post‐traumatic stress disorder (PTSD). I do not, however, intend to pathologize Persephone: her anguish needs not be labelled. It is just what it is—suffering—even if her symptoms find a place in the DSM–5 (Diagnostic and Statistical Manual of Mental Disorders, Fifth Edition). I argue that Persephone’s predicament is so contracting as to not allow for any expansion, let alone rebirth: Rape condemns Persephone (and her mother Demeter) to a life of traumatic repetition which eventually leads to destruction. Even the goddess Demeter, despite all her powers, is unable to undo her daughter’s rape and bring her back from death (Rich, 2021).

I begin by examining Patricia Berry’s (2008, 2013) reading of the Demeter/Persephone myth, particularly her concept of *rape into consciousness*, exploring how her symbolic approach illuminates internal dynamics within the individuation process. 1 While Berry’s concept offers a compelling metaphor for the ego’s sometimes violent experience of coming into consciousness, I contend that this symbolic approach could potentially *shield* us from the brutal reality of *literal* rape, a reality the Homeric *Hymn to Demeter* encapsulates.

Jung’s concept of *recalcitrant fourth* offers a crucial counterpoint, demanding a shift from the symbolic to the empirical world. This violent flight back to ground forces a confrontation with life’s material and sensorial dimensions, where literal rape reaps trauma and death. Jung’s fourth, reconceptualized here as rape into consciousness, compels the retrieval of the *neglected*, that is, the elements missing from conscious awareness.

Guided by this concept and the Persephone myth, I turn my attention to the concrete aspects of rape, beginning with the events of October 7^th^. Interspersed throughout my analysis is Glück’s (2021) haunting song to Persephone, which serves as both a poignant elegy for the victims and as a calming tune for me. Glück’s powerful portrayal of Persephone’s trauma not only offers a profound reflection on the myth but also illuminates my own understanding of its enduring relevance.

The Persephone/Demeter myth, according to Jung, alludes to a split between matriarchal and patriarchal worlds. The Homeric *Hymn* intensifies this fracture, bringing into full view the violence perpetrated by the patriarchy against the two female protagonists. Played out in the domain of gods and goddesses, this archetypal gender conflict finds its echo in the human sphere, across a spectrum of experiences, from the intimacy of domestic life to the brutality of war and terror, with the events of October 7^th^ providing a stark and recent example.

## Rape and the Symbolic

“To know the psyche at its basic depths, for a true depth psychology, one must go to the underworld” (James Hillman, 1979)

In her seminal work, “Neurosis and the rape of Demeter/Persephone”, Berry introduces the concept of “rape into consciousness” to describe a particular kind of traumatic awakening (2008, p. 28; 2013, p. 204). This potent metaphor emphasizes the sometimes violent and disruptive nature of the ego’s journey toward self‐awareness. Rape in this case is conceived as an initiation into the realm of psyche, an inevitable intrapsychic phenomenon inherent to the process of individuation.

Berry, who views Persephone as a facet of Demeter (Downing, 2013), 2 reads the Demeter/Persephone myth in terms of the reorientation of an ego which the author considers excessively one‐sided: Persephone is too naïve, and Demeter too superficial. In these cases, says Berry, a movement towards introversion, the descent into hell, becomes necessary and it is therefore activated in the unconscious. Persephone must be surrendered to the underworld, 3 and Demeter must find a way to access its profundity, her daughter’s dwelling. Demeter, in other words, “needs the underworld experience into which Persephone initiates her—that is what she is seeking as she engages in her search for Persephone” (Downing, 2013, p. 187).

Demeter’s search for her daughter/psyche, however, veers into a manic quest which, according to Berry, displays all the signs of pathology. In Berry’s words, the goddess becomes “an example of a mythic figure evidencing neurotic behavior” (2008, p. 17). Demeter, Berry goes on, feeds her own neurosis by avoiding the vertical descent into the depth of the archetype. Suffering in her case becomes a destructive, if defensive measure to evade the inevitable encounter with the archetypal realm of the underworld, a dissociation from the divine depths which Berry describes as being “very destructive” (2008, p. 24). Indeed, rather than moving downwards, the goddess seeks refuge “in the polis, the world of everyday events, ‘reality’” (p. 23).

Whilst Demeter cannot let go of the upper world, resisting descent, Berry appears to pathologize earthly reality. In her account of the myth, the upper world becomes the domain in which neurotic repetition cannot be escaped. For Berry the cure, if any, rests on psychic rape, the violation of Demeter/Persephone’s consciousness. Rape is a horror, says Berry, “but when and however it is constellated, if it connects to the Demeter/Persephone archetype, then the violation is not only possible but essential” (p. 26).

Naturally, as James Hillman (1979) suggests, there are less traumatic avenues to the archetypal realm; however, writes Christine Downing, “there is a particular style of consciousness … that resists other modes of descent to underworld experience and so constellates rape, the forced descent” (2013, p. 187). In the myth, Berry argues, this resistance draws “rape and violence upon itself” (2008, p. 25). As Hillman comments, we are raped into the underworld “only if we are out in Demeter’s green fields, seductively innocent with playmates among flowers” (1979, p. 49).

## Normalizing Rape?

The symbolic approach to rape, exemplified by Berry’s and Hillman’s interpretation of the Demeter/Persephone myth, offers valuable insights into the workings of the internal world. However, one may argue that giving priority to the symbolic‐archetypal aspect of rape over its literal counterpart risks normalizing the specific reality of *actual* rape. This approach, in other words, may inadvertently contribute to what feminists have identified as a desensitization to the horrors of aggravated gender‐based violence in Western culture.

A significant factor contributing to this desensitization is the pervasive presence of rape in Greco‐Roman mythology. Feminists contend that this abundance has normalized “sexual violence and rape culture in the West” (Judge, 2024, p. 19; Rabinowitz, 2014; Morales, 2020). Jung himself acknowledges the ubiquity of rape in mythology, noting that it frequently recurs as a theme: “One has only to think of the rape of Persephone, of Deianira, Europa, and of the Sabine women” (1912/1952, para. 34).

Feminists may indeed have a point: Shouldn’t we be perturbed, for instance, by the choice of Rembrandt’s “Abduction of Europa” (the myth is also known as “The Rape of Europa”) for the cover of *Europe’s Many Souls,* a fairly recent publication edited by two renowned Jungians, Joerg Rasche and Thomas Singer? One of the least obscene accounts of Europa’s abduction, according to classicist Helen Morales, describes the scene as follows:

Zeus saw Europa, the daughter of Phoenix, when she was gathering flowers in a meadow along with some nymphs, and he desired her. He came down and changed himself into a bull and breathed saffron breath from his mouth. In this way he deceived Europa, carried her off, took her to Crete, and had sex with her. (2007, p. 6)

In what way is this depiction of rape entering a discussion on cultural complexes, identities, and group relations? While the representation of Europa’s rape could allude to the “serious fractures in the dream of European reconciliation and unity” (Singer & Rasche, 2016, p. 2), the image fundamentally depicts a violent sexual assault. Does the use of this imagery indicate a desensitization to the brutal realities of actual rape?

Another issue I see with granting priority to the symbolic is that earthly reality (as opposed to archetypal reality) comes to be associated with that which is deemed to be problematic: ego‐consciousness, pathology, neurosis, lack of soul, etc. Working at the Demeter level of experience, the ego is dismissed as “the great literalist, positivist, realist” (Hillman, 1979, p. 64). Psyche, on the other hand, is placed firmly in chthonic depths of the archetypal realm, separated from the upper world, the world of ordinary events.

One can argue that, when overplayed, this Jungian focus on symbolic‐archetypal themes could easily veer into what Polly Young‐Eisendrath calls the “Jungian folly”: a tendency to “swim around in the world of archetypal imagery,” stressing the “restorative aspect of unconscious reality” (2013, p. 209). 4 Jung, himself prone to this kind of folly, intuited the risk of his own psychology losing the grip on the everyday facts of life. He clearly saw this danger reflected in Plato, whose life Jung criticised for lacking earthly grounding. “What sort of philosophy would Plato have produced had he been his own house‐slave?”, Jung asks (1948, para. 264). Ironically, Jung’s critique of Plato’s detachment from reality mirrored his own tendency to downplay the significance of the mundane and the material.

Jung acknowledged prioritizing the symbolic/archetypal world over the perceived triviality of everyday existence. For example, in a letter dated 1932 addressed to Christiana Morgan, a former patient, he declared: “Life on a personal level is the smaller affair, the higher level however is impersonal” (cited in Saban, 2019, p. 219). This sentiment was echoed in a 1939 lecture where Jung further emphasized:

Now, we have no symbolic life, and we are all badly in need of the symbolic life. … And because people have no such thing, they can never step out of this mill—this awful, grinding, banal life in which they are “nothing but”. … Everything is banal, everything is “nothing but”; and that is the reason why people are neurotic. They are simply sick of the whole thing, sick of that banal life … 5 (para. 627)

In 1945 Jung reiterated that the “approach to the numinous” was his primary area of interest (1973, p. 377). He justified this shift in focus away from the “treatment of neuroses” by arguing that engaging with the numinous could help individuals overcome the “curse of pathology” (p. 377).

In truth, Jung’s life and work displayed greater balance. However, as Mark Saban observes, and as these excerpts demonstrate, Jung’s psychology, in its mature form, prioritized the symbolic to the detriment of “outer relationships, the social, the collective” (2019, p. 136). This compartmentalization, though, falls apart with the fourth, as the soul becomes rooted in the “most ordinary, broken, and seemingly inconsequential moments” (Lamborn, 2015, p. 38). For, as Amy Lamborn puts it, “it is particularly in these kinds of possibilities that Jung’s fourth does its work” (p. 38). Individuation, Lamborn goes on, necessitates engaging with the “earthy, the banal, the excluded”—all that remains outside our conscious awareness and often contradicts our highest ideals (Lamborn, 2015, p. 42).

## The Recalcitrance of the Fourth

Jung’s recalcitrant fourth remains an unfamiliar concept to many in the Jungian community. Left undeveloped by Jung himself, intuited yet not properly defined, this notion appears to serve as a corrective to the potential shortcomings of Jung’s mature psychology which, as noted, often tends to devalue everyday experiences as banal and insignificant. 6

Jung (1948) considered the recalcitrant fourth as he explored the psychological meaning of the trinitarian symbolism in his essay “A Psychological Approach to the Dogma of the Trinity”. Presenting it as feminine, material, or evil, Jung positioned the fourth in stark opposition to what he characterized as the purely masculine, luminous, and benevolent qualities of the Trinity. It is this oppositional stance that makes the fourth *recalcitrant*. Indeed, as Wolfgang Giegerich (2020) observes, recalcitrance is the very essence of the fourth: The fourth is inherently adversarial.

In the Trinity essay, to substantiate his theory of the fourth, Jung intriguingly drew upon the cryptic opening of Plato’s *Timaeus*, boldly asserting that “[e]ver since the *Timaeus* the ‘fourth’ has signified ‘realization’, i.e., entry into an essentially different condition, that of worldly materiality” (1948, para. 251). However, as noted by Giegerich (2020), earthly realization was not the aim of either Plato or the *Timaeus*, but rather that of Jung himself. 7

Giegerich suggests that the fourth represents Jung’s “yearning for actuality” (p. 119), a potential desire for grounding his psychological theories in the messy reality of human affairs and social realities. Here, the tension between harmony and its disruptive forces is played out on a grand scale. While a full exploration of Giegerich’s in‐depth critique of Jung’s concept is beyond the scope of this paper, it is important to note Giegerich’s strong objection to what he terms Jung’s “psychological *materialism*” (p. 153, italics in original), exemplified by Jung’s theory of the fourth. He argues that engaging with the mundane everyday world—via the fourth—threatens to undermine the core of psychology, a discipline fundamentally concerned with the inner world. Giegerich sharply criticizes Jung for this apparent deviation from the discipline’s central focus.

Jung’s fourth is concerned with the material world, concrete reality, and especially its neglected aspects. As Murray Stein observes, the fourth “indicates the critical presence (*or absence*) of ‘reality’ in physical, political, social, down‐to earth terms” (1985, p. 116, italics mine). Similarly, Ann Ulanov characterizes the fourth as “[a]ll the stuff, the *materia prima* that does not get included in conscious living, both personal and communal (2007, p. 593, italics in original). Ulanov points to the continued exclusion of the feminine from conscious life, despite feminist progress. She defines the feminine as a mode of being inherent to all genders, encompassing: “grounded, not abstract knowledge, embodied wisdom, not generalities, the earth of daily life, not the heaven of a next life, the mixture of affect and idea, poetry and concepts, the universal through the local, particular and personal” (p. 594).

The fourth, thus, embodies the neglected—that which is felt but remains largely unintegrated. However, the fourth might also be conceptualized as a psychic factor exerting a dynamic pressure towards the recognition and potential integration of elements that are otherwise unassimilable. This perspective is corroborated by Ulanov’s analysis, which identifies the fourth as that which “prompts us to see,” or that which “is engineering us to include all the parts of ourselves” (2007, p. 594).

Echoing Berry’s notion of rape into consciousness as explored in her interpretation of the Persephone myth, the fourth points to the need for ego realignment, suggesting an archetypal constellation that violently destabilizes the ego. For its part, the ego perceives this movement as a terrible violation, indeed as a rape. Reconceptualized in this context as a rape into consciousness, the fourth forces a return to the sensory and material world, where literal rape inflicts trauma and death.

## Back to Ground: Literalizing Persephone’s Myth

I wish to talk about literal rape, with specific emphasis on the gang‐rape of Jewish women that occurred on October 7^th^, 2023. I will explore these violent acts through the lens of the myth of Persephone, as told in the Homeric *Hymn to Demeter*. Literalizing the Persephone myth, treating Demeter and Persephone as though they were real persons, could be seen by Jungian scholars as a category error. Yet, one can argue that the strength of a myth resides in its ability to resonate with our lives, and many a woman has identified with the traumatic experience the myth encapsulates. As Downing writes in her introduction to *The Long Journey Home: Revisioning the Myth of Demeter and Persephone for Our Time*, there is a social dimension to the myth that is “pertinent to the lives of contemporary women” (2013, p. 2). Further, commenting on one of the essays included in the collection, Downing remarks: “In a culture where rape is an everyday actual reality, Hades’ rape of Persephone is read not just as a metaphor but as a reference to the very real vulnerability of women” (p. 147).

Indeed, according to classical scholar Nicholas Richardson, this particular myth stands out not only because of the “sustained intensity and dramatic power of its narrative,” but also because of the “seriousness of its theme, the rape of Demeter’s daughter Persephone by the lord of the underworld and its consequences” (2011, p. 44). What is unusual about this myth, according to Richardson, is that the “focus of attention is on the female characters, both divine and human” (p. 47). 8 The poet encourages us to identify and empathize with these figures, and especially with “Demeter and Kore in the portrayal of their suffering” (p. 47).

But the anguish of the two female protagonists is not the sole focus of the story. What the myth wants us to see and engage with is, I believe, the tension between matriarchal and patriarchal orders of experience, a tension the myth brings to light. Jung suggests this much in “The Psychological Aspects of the Kore”. In this essay Jung cautions that the “Demeter‐Kore myth is far too feminine” (1951, para. 383), and as such difficult for men to fully comprehend (Downing, 2013): “Demeter‐Kore exists on the plane of mother‐daughter experience, which is alien to man and shuts him out,” Jung writes (1951, para. 383). 9 Jung also observes that the Eleusinian mysteries associated with the myth convey the image of a matriarchal society at odds with its male counterpart.

In the *Hymn,* we see this gender conflict unfolding: The misogynistic fantasies of the male characters are acted out, with Persephone, the object of Hades’ desire, being brutally forced into the underworld, raped, and killed. Objectification, brutality, misogyny, murder, and the underworld in the guise of tunnels, all recur on October 7^th^ in a disturbing re‐enactment of the myth’s own configuration.

## October 7^th^: The Rape

“There is in the words ‘a beautiful Jewess’ a very special sexual signification. … This phrase carries an aura of rape and massacre” (Jean‐Paul Sartre, 1948/1995)

The all‐male enemy commando came by land, air, and sea. Filming their actions, the men stormed the Supernova music peace festival and *peacenik kibbutzim* on the holy day of *Simchat Torah*. They ravaged the young women in a savage frenzy of sadism, power, and terror. It was systematic gender‐based violence; it was femicide.

death cannot harm me
more than you have
harmed me,
my beloved life.
(Glück, 2021, p. 9)

Many were killed, disfigured, tortured, burned, and mutilated: they played with their breasts after cutting them off. In the morgue, the women in charge of preparing the bodies for burial saw broken pelvises, clenched fingers, dislodged legs, charred limbs, distorted faces. 10

death cannot harm me
more than you have
harmed me,
my beloved life.

Shani Louk was brought back in a pick‐up truck to the men’s enclave to be paraded half naked as a spoil of war. Men on the street spat on and bit the lifeless body, whilst another sat with his boot on her back. A large fragment of Shani’s skull was later found at the site of the Supernova festival. She was declared dead at this point.

death cannot harm me
more than you have
harmed me,
my beloved life.

Naama Levy is filmed by the attackers: she is limping, barefoot, blood darkens the fabric at her groin, her hands tied behind her back, her ankles cut. Looking resigned to her fate, Naama does not react as she is forced into the back of a Hummer.

death cannot harm me
more than you have
harmed me,
my beloved life.

In another video, a modern Kore/Persephone, Noa Argamani, is seen screaming “with a shrill voice” (Hom*. Hymn Dem*. 20), just as in the Homeric *Hymn to Demeter*, pleading with her abductors “out at the top of [her] voice” (Hom*. Hymn Dem*. 432). Despite her cries, she is overpowered and snatched away. 11

death cannot harm me
more than you have
harmed me,
my beloved life.

The protagonist of Louise Glück’s *Averno* (Gosmann, 2010; Azcuy, 2011), Glück’s Persephone is described as a drifter between worlds: not completely dead, not entirely alive.

## Glück’s Persephone

Averno, west of Naples, is a small volcanic lake believed by the Romans to be the entrance to the underworld. In the *Aeneid*, Virgil describes *Avernus* as an “evil‐smelling throat” (*Aen*. 6.202), a cave around which no “bird could wing its flight … , so deadly was the breath that streamed out of that black throat and up into the vault of heaven” (*Aen*. 6.239–242). 12

Beginning with “October”, 13 at “the inception of the dying season” (Azcuy, 2011, p. 33), Glück’s *Averno* brings together the mythical, the personal, and the historical.

This is the light of autumn, not the light of spring.

The light of autumn: *you will not be spared*. (Glück, 2021, p. 11, italics in original)

The mythical reflects the horror of the abduction, rape, and murder of Demeter’s daughter Persephone, whose fate is sealed by the patriarchy: her father Zeus and her uncle Hades, aided by Gaia who, says Marilyn Arthur, “cooperates in the scheme to assert male dominion” (2013, p. 222).

Kore, a not yet Persephone, is picking flowers in the lush meadow accompanied by other virgins when, 14 appearing from the earth’s depth, Hades shocks the maiden into submission, forcefully delivering her to the world below where he resides. 15 There, according to the Homeric *Hymn*, the Lord of the Dead bullies the goddess into ingesting a pomegranate seed (412–413), thus making young Kore his death‐bride, Persephone.

Tell me this is the future,
I won’t believe you.
Tell me I’m living,
I won’t believe you.
(Glück, 2021, p. 8)

The *Hymn* highlights the wonderous (narcotic) effects the narcissus has on the maiden. 16 Indeed, according to ecofeminist Gloria Feman Orenstein, the flower is part of the “snare that Zeus had connived in order that she be abducted and married off to Hades … against both her will and that of her mother.” Orenstein goes on: “Thus, I see that Persephone was drugged/poisoned, abducted … sacrificed to the god of the underworld, and raped (of course, since this all took place contrary to her will)” (2013, p. 262). Glück makes a similar point as she mocks the perverse rape fantasy of the scholars who debate on whether Persephone instigated the rape or not (Gosmann, 2010):

did she cooperate in her rape,
or was she drugged, violated against her will,
as happens so often now to modern girls.
(Glück, 2021, p. 16)

Persephone is now Queen of the Underworld: rape, whose aim is objectification and dehumanization, has killed her. “What can I say?” utters Briseis in *The Silence of the Girls*, a feminist adaptation of the *Iliad*, Achilles “fucked as quickly as he killed, and for me it was the same thing. Something in me died that night” (Barker, 2019, p. 28).

*What can I say*? “Narrative requires a narrator,” writes anthropologist Roberta Culbertson, “but the destruction of the self at the root of much violence makes this narrative nearly impossible by definition. The question is not only ‘what is there to say’ but ‘who is there to talk?’” (1995, p. 191). What, then, is left of Persephone?

“The victim of incest and parricide” (Azcuy, 2011, p. 37), Persephone is transformed into a spectral creature inhabiting the “traumatic void” (Caruth, 1995, p. 7) of the land of the dead, to which she is destined to return cyclically. “Stained with red juice” (Glück, 2021, p. 16), Persephone is released “from the misty gloom into the light” (Hom. *Hymn Dem*. 337–338), back to Demeter, only to be forced to leave the upper world again when called to her duties as Queen of Hell. Her soul split more than she can bear, Persephone is destined to be a drifter in both the realm of the living and that of the dead (Gosmann, 2010).

You do not live;
you are not allowed to die.
You drift between earth and death
which seem, finally,
strangely alike.
(Glück, 2021, p. 18)

Rape has locked Persephone (and her mother) into an endless cycle of traumatic repetition, a symptom of PTSD: she is, in effect, *PTSdead*.

## PTSdead Persephone

Trauma specialist Cathy Caruth describes PTSD as:

a response, sometimes delayed, to an overwhelming event or events, which takes the form of *repeated*, intrusive hallucinations, dreams, thoughts or behaviors stemming from the event, along with numbing … and possibly also increased arousal to (and avoidance of) stimuli recalling the event. (1995, p. 4, italics mine)

As the experience is not fully assimilated at the time of the actual incident(s), repetition becomes an “attempt to master what was never fully grasped in the first place” (Caruth, 1996, p. 62). This literal, non‐symbolic return to the event(s) against one’s will, is what makes survival almost an impossibility. And indeed, for Caruth this “determined repetition of the event of destruction,” characteristic of the history of the traumatized individual, attests to the “impossibility of living” (p. 65). From this perspective, writes Caruth:

the survival of trauma is not the fortunate passage beyond a violent event, a passage that is accidentally interrupted by reminders of it, but rather the endless inherent necessity of repetition, which ultimately may lead to destruction. (pp. 62–63)

Persephone’s cyclical return to the underworld could thus be interpreted as a repetition of the death‐like break brought about by the violence of rape: “violence has changed me,” laments Glück’s Persephone, “[m]y body has grown cold like the stripped fields” (2021, p. 7). Violence, writes Culbertson, “is about … dissolution,” and the body, Culbertson continues, becomes the “reference point … of all power relationships sustained by violence,” especially since the victim will come to “experience her body in the way dictated by the perpetrator” (1995, p. 172).

Sociologist Jane Kilby provides an insightful critique of Culbertson’s essay. Kilby is troubled by Culbertson’s inclination to romanticize rape, portraying it as a transcendent experience of rebirth. Whilst for Culbertson “survivors are unwilling, uninitiated, unprepared, unschooled mystics” (1995, p. 178), for Kilby there is nothing to suggest that rape is a “gift holding the promise of self‐knowledge and enlightenment” (2008, p. 106). The trauma of violation does not tell much to the initiated, who experiences the outside as brutally invading the inside without the mediation of consciousness. Rape breaks the ego into fragments, collapsing its ability to understand and integrate an experience that has escaped its own boundaries and as such cannot be reclaimed as one’s own (Caruth, 1995). In a recent essay, Monica Luci captures the devastating impact of this boundary dissolution, describing it as a “profound rupture in the ‘psychic skin’” (2024, p. 775). “When the psychic skin no longer offers protection”, Luci explains, the “psychic body” inevitably “fragments” (p. 775). 17

This inability to register one’s own trauma is what Dori Laub (2013) calls the “collapse of witnessing,” a condition in which the insider is so contaminated by the power of the event as to be unable to witness it. It is this lack of assimilation that condemns the individual to the endless repetition characteristic of PTSD. PTSD, however, is not the way in which Persephone’s journey is generally understood. Scholars have interpreted the abduction, rape and killing of young Kore as a coming‐of‐age story, the passage from maidenhood to womanhood culminating in rebirth.

## Born‐again Persephone? No.

Indeed, Professor of *Classics Helene* Foley explains that:

Persephone’s experience of abduction, symbolic death, and rebirth into the upper world could be associated with the transition or initiation of women into marriage. The myth’s enforced separation of mother and daughter followed by reunion was celebrated in ancient cults special to women and seems to reflect, on the psychological level, the pattern of maturation common to mothers and daughters cross‐culturally. (2013, p. 96)

Persephone’s myth, her abduction and rape are thus thought to signify an initiation into the realm of womanhood through marriage. Persephone “will never be Kore, the maiden, again. She has matured, become sexualized, died, and been reborn” (Lincoln, 2013, p. 172). Yet, the myth also registers the fierce reaction of the two goddesses against the forceful breaking of the matrilinear connection, 18 a breach so violently rejected by Demeter as to drive her into direct conflict with the patriarchy. Indeed, “Demeter directly challenges the patriarchal reign of Zeus and comes within a hair of entire success” (Foley, 2013, p. 80). Unfolding in the divine realm, this antagonism, writes Carl Kerényi, sees Persephone’s connections with mother and husband “carried to the extremes and balanced against one another. One of the forms (daughter with mother) appears as life; the other (young girl with husband) as death” (1951, p. 127).

Jung acknowledges that the “the psychology of Demeter cult”, the Eleusinian Mysteries associated with the myth, “bears all the features of a matriarchal order of society, where the man is an indispensable but on the whole disturbing factor” (1951, para. 383). In his analysis of the Demeter cult, Neumann reaches a similar conclusion: “[I]n the eyes of this female group, the male is an alien who comes from without and by violence takes the daughter from the mother” (1955, p. 479). Foley makes a similar point as she submits that the myth speaks of a gender conflict that, playing out in the archetypal plane of gods and goddesses, attempts to counter the misogyny of the patriarchal order. The *Hymn*, writes Foley, “develops its narrative on the divine level as a conflict between genders. Demeter, Hekate, and Persephone are aligned on one side: Zeus, Helios, and Hades on the other” (2013, pp. 104–105). This archetypal gender conflict is replicated in the human sphere in its endless variations. From time immemorial women have been abducted, sexually violated, and symbolically or actually murdered: their matrilinear connection (mothers and daughters) disrupted by men. Demeter herself, all too human in her sorrow, 19 recounts how she was made the subject of violence as she was abducted by pirates in Crete. The story is an invention of the goddess; however, the account is false only on the surface, “for the message of the tale is true. Demeter tells of enforced submission to men and enslavement to their desires” (Arthur, 2013, p. 227):

On the broad back of the sea I have come now from Crete,
by no wish of my own. By force and necessity pirate *men*
led me off against my desire.
(Hom. *Hymn Dem*. 123–125; italics mine)

In mentioning the seizure of Demeter by pirates, the *Hymn* hints at something that goes beyond the confines of domestic life and marriage, as piracy was part of early Greek warfare as much as pitched battles, incursions, ambushes, and guerrillas (Lloyd, 2017). The *Hymn* is not an historical document; it is however a literary document, and when it comes to archaic Greece historians seem to be using these ancient texts as a source. They agree, for instance, that “Homer’s description of the mythical siege of Troy in the *Iliad* provides crucial information about the earliest Greek attitudes towards civilian involvement in war” (Williams, 2018, p. 26).

What we evince from these ancient sources is that whatever the tactic of war, the fate of the many Persephones involved in conflict was the same: they were either gang‐raped and killed *in situ* or captured and sexually enslaved. 20

## Wartime Persephone

“Of all the horrors [of war], the rape of women is the worst”. 21 (Scholium to the *Iliad*)

The ultimate intent of rape in ancient warfare was that of eliminating a social group, its laws and culture. As a genocidal tool, mass rape left the enemy population in ruins.

In the *Iliad*, the violence suffered by women during and after the military campaigns is barely disguised by euphemisms such as sleeping with, lying next to, bedding down with (Gaca, 2018). The command to rape the women of the enemy camp came from the top and had to be fulfilled by combatants or else face death. Nestor orders his troops to avenge Helen’s suffering by raping all the women they could lay their hands on: “Let no one be eager to return home until he has slept with a wife among the Trojans” (*Il* 2.354–356, cited in Gaca, 2018, p. 59).

Historian Kathy Gaca’s investigation of gender‐based violence in ancient warfare attests to the brutality of the practices inflicted on women by the enemy forces. The assailants believed that the systematic rape, murder, or seizing of women was a justifiable and fair objective of war, “a view with which the devastated survivors … understandably disagreed, to the extent that their perception is registered historically” (Gaca, 2010–2011, p. 86). Groupthink allowed the aggressors to see themselves as superior to the opponent who they regarded as deserving the most horrific of treatments, especially when it came to women.

According to Gaca, one of the purposes of heterosexual gang‐rape, particularly that of freeborn women and girls, was that of replacing civil law with a martially regulated system which saw female captives treated as “she‐animals to be broken in, beaten” and sexually forced into submission (p. 89). Their body and spirit shattered, these women found themselves cut off from their kin group and bereft of their former social and ethnic identity, a fate usually shared by their progeny. Ultimately, genocide was effected through sexual enslavement and the violent take‐over of the women’s “reproductive lifeline” (p. 106).

There is, sadly, a remarkable correspondence between these ancient practices of populace‐ravaging warfare and the genocidal rapes of the Persephones of our times, including those murdered or sexually abused during and after October 7^th^. The correspondence between ancient and modern‐day events speaks of a historical continuity which sees humanity as perpetually linked, on a collective level, to its ancient past (Lu, 2011). 22 Saturated with misogynistic fantasies and brutality, the archetypal pattern of the Demeter/Persephone myth keeps on replicating itself in the human plane, with the patriarchy still capable of ravaging the female body, inflicting unspeakable sorrow on the daughters and mothers of this battered world.

## Conclusion

Berry’s interpretation of the Demeter/Persephone myth, and her concept of rape into consciousness, sheds light on the dynamics of the intrapsychic world. However, this symbolic lens may inadvertently distance us from the devastating reality of literal rape—a reality captured by the myth itself. In contrast, Jung’s recalcitrant fourth insists on a move from the symbolic to the empirical: a violent flight back to ground, to the sensory and material dimensions of lived experience, where literal rape inflicts trauma and death.

Reconceptualized here as rape into consciousness, the fourth demands the recovery of the neglected: those elements absent from conscious awareness. Using this concept and the myth of Persephone, I explored the real‐world realities of rape, urging the reader to witness its traumatic consequences.

For Jung, the Persephone/Demeter myth symbolizes the split between matriarchal and patriarchal worlds. The Homeric *Hymn* intensifies this division, bringing into sharp relief the violence committed by the patriarchy against the two female characters. Occurring in the divine realm, this archetypal gender conflict reverberates throughout human experience, appearing in many forms, from personal to global, including the horrors of war and terror. The events of October 7^th^ serve as a grim reminder of its continued relevance.

## Footnotes

^1^
In a recent autoethnographic article (Cerminara, 2025a), I apply Berry’s concept of rape into consciousness to examine my own descent after the October 7^th^ massacre.

^2^
Similarly, Jung sees the pair Demeter‐Persephone as aspects of each other: “We could therefore say that every mother contains her daughter in herself and every daughter her mother, and that every woman extends backwards into her mother and forwards into her daughter” (1951, para. 316).

^3^
The underworld is psyche, says Hillman (1979, p. 46).

^4^
Young‐Eisendrath’s warning is echoed by Mark Saban. He writes: “What Jung doesn’t seem to be willing to entertain is the possibility that one might also become ‘spellbound’ by an archetypal realm, untethered to the personal. In both cases, to be spellbound means to be one‐sided” (2019, p. 221). Saban also points out that by according prominence to the archetypal realm over the social and political ones Jung is in fact doing a disservice to his own psychology, whose main tenet is the process of individuation.

^5^
Jung, throughout the lecture, displays a profound unease with what he perceives to be the banality of everyday life. He repeatedly employs the term “banal” to express his disdain for the mundanity of common human experiences, such as career pursuits and the “producing of children” (1939, para. 630). As a member of the audience expresses concern that a detachment from the material world might encourage individuals to escape the demands of everyday life by taking refuge in symbolism, Jung concedes that those who intentionally isolate themselves from the realities of daily existence could be considered “cheats” who misuse the symbolic life as a form of escapism. However, he intriguingly notes that these individuals often “can stand it somehow without getting too neurotic” (para. 649).

^6^
I have explored Jung’s notion of the recalcitrant fourth in two previous articles (Cerminara, 2024, 2025b).

^7^
Alongside Giegerich, Paul Bishop (2022) offers a masterful analysis of Jung’s interpretation of Plato, pointing out Jung’s selective and self‐serving reading of the *Timaeus*. Jung, in Bishop’s words, attempted to convert “Plato’s cosmology into the terms of his *own* psychology, and whether or not this succeeds on *conceptual* grounds is, for him, less important than whether it succeeds on *clinical* or *therapeutic* grounds” (2022, p. 82, italics in original).

^8^
The *Hymn*, according to Arthur, reveals a “peculiarly feminine sensibility” (2013, p. 215). Arthur goes on to write that the *Hymn* “treats the transition from ‘matriarchy’ to ‘patriarchy’ from the female point of view and therefore takes the form of a series of attempts to resist male domination” (p. 216).

^9^
Downing argues that in this essay “Jung recognizes the difference between the anima, the unconscious feminine aspect of men, and the psyche of women themselves and consciously struggles to honor the difference more than in perhaps any other of his writings” (2013, p. 68).

^10^
According to *The Lancet* (November 30, 2023, https://www.thelancet.com/journals/lancet/article/PIIS0140‐6736(23)02654‐5/fulltext), elements of the violence inflicted on women on October 7^th^, which involve “reproductive degradation, including mutilation of breasts”, reflect “a systematic assault recognized as a form of genocide by the Rome Statutes” (p. 2290). Interestingly, testimonies from the Lvov pogroms of 1941 describe similar cruelties, including the cutting of breasts (see: Levenkron, 2010, pp. 17–18). The slicing of women’s breasts is a recurrent practice in history as attested by ancient sources such as Herodotus (Gaca, 2010).

^11^
All my citations from the Homeric *Hymn to Demeter* are derived from Foley (2013) and make use of the author’s translation.

^12^
Virgil, *The Aeneid*. Penguin Classics, David West’s (2003) translation.

^13^
This paper is particularly indebted to Mary Kate Azcuy’s (2011) article.

^14^
In Greek myth meadows are “liminal sites, associated not only with a transition to sexuality and fertility but with the underworld” (Foley, 2013, pp. 33–34).

^15^
It is not clear whether sexual violence takes place at this point, but the *Hymn* clearly registers the brutality of the attack: The maiden is overpowered by force and suffers violence at the hand of her assailant (Foley, 2013).

^16^
Kerényi writes: “Poets have never failed to catch the significance of this scene. For one of them [D. H. Lawrence] the flowers were ‘hell‐hounds on her heels’” (1951, p. 128).

^17^
“The boundaries of my body are also the boundaries of my self,” writes Jean Améry in his seminal 1966 essay on torture. “My skin surface shields me against the external world. If I am to have trust, I must feel on it only what I want to feel. At the first blow, however, this trust in the world breaks down” (1980, p. 28).

^18^
Writer Vera Bushe (2013) interprets Demeter’s excessive reaction in terms of a necessary breaking of the narcissistic bond between mother and daughter.

^19^
Demeter, Kerényi writes, “leads us back to something purely human” (1951, p. 134).

^20^
Gang‐rape, writes Gaca (2010–11) was limited to women, who were stripped naked and shamed before an all‐male audience.

^21^
Cited in: Gaca (2018, pp. 59–60).

^22^
In “The Role of the Unconscious”, Jung distinguishes between what he calls “natural history” and “objective history” (1918, para. 12). A clearer distinction is proposed by Kevin Lu who suggests the use of a different terminology: “archetypal history” and “conscious history”. Archetypal history is an inherited component of the human species. “This form of history,” Lu explains, “dwells in the unconscious, and only becomes partially distinguishable through its manifestations as conscious history” (2011, p. 16).
